# Suggestion of Safety Certification Standards and Performance Evaluation Methods for Fabricated Mobile Scaffold in South Korea

**DOI:** 10.3390/ijerph19010133

**Published:** 2021-12-23

**Authors:** Heesoo Kim, Jeonghyeon Lim, Jeong-Hun Won, Jun-Hyuk Kwon, Seungjun Kim

**Affiliations:** 1 School of Civil, Environmental and Architectural Engineering, Korea University, Seoul 02841, Korea; kf99403@korea.ac.kr (H.K.); bestcivileng@korea.ac.kr (J.L.); 2 Department of Safety Engineering, Chungbuk National University, Cheongju 28644, Korea; jhwon@chungbuk.ac.kr; 3 Temporary Equipment Certification Department, Occupational Safety & Health Certification Institute, Ulsan 44429, Korea; bandit@kosha.or.kr

**Keywords:** construction safety, fabricated mobile scaffold, load-carrying capacity, safety certification standard, temporary equipment

## Abstract

At construction sites, various types of temporary equipment and structures are used for safety and work efficiency. However, various temporary equipment-related accidents frequently occur for many reasons, including inappropriate installation, usage, and material and structural imperfections. A mobile scaffold is one of the most commonly used indoor temporary equipment for work in high places. In general, the main structural members of the mobile scaffold, such as the mainframes, horizontal members, braces, caster wheels, outriggers, and handrails, are installed on the construction site for this purpose. This means that the load-carrying capacity of the equipment can vary depending on the assembly details. In Korea, there are safety certification standards applied for frequently used temporary equipment, such as scaffolds and shoring. However, the standards concern the strength criteria for the member itself, rather than the global load-carrying capacity. Therefore, it is difficult to review whether the fabricated mobile scaffold has sufficient load-carrying capacity, or to confirm the structural safety considering the various uncertainties affecting the structural performance. In this study, rational safety certification standards and evaluation methods are suggested for fabricated mobile scaffolds. The suggested safety certification standards present structure-level criteria for checking the load-carrying capacity, horizontal stiffness of the structure, and overturning risk. It is expected that the structural performance for safety can be directly checked based on the suggested safety certification standards and performance evaluation methods during the safety certification stage.

## 1. Introduction

Temporary structures are facilities installed for the construction of buildings or structures in construction work; when construction is completed, they are dismantled and demolished. In general, “temporary structures” denotes scaffolds, casts, sheathing timbering, etc. Temporary structures may have reduced strength and/or structural defects. Therefore, the materials and structural shapes of the members used, connection conditions, and assembly method should be carefully considered, particularly to the risk of serious disasters owing to collapse or overturning.

When a structural defect exists in a temporary structure, a collapse accident may occur, greatly threatening the safety of workers. Therefore, it is important to ensure product safety. Accordingly, many countries have suggested materials, structural standards, and structural performance standards for temporary structures, and manufacturers have been required to design and manufacture products that comply with them.

According to the United States Bureau of Labor Statistics (BLS) [1], scaffold-related accidents result in approximately 60 deaths and 4500 injuries every year. Approximately 25% of the falls from scaffolds (for all working surfaces) are fatal. According to a recent BLS study, 72% of scaffold accidents are attributable to one of the following three causes: scaffold support or planking problems owing to defective equipment or improper assembly, slipping or tripping while on a scaffold owing to factors such as slippery surfaces or a lack of handrails, and falling objects hitting either a worker standing on a scaffold or workers below. The remaining 28% of accidents are caused by electrocution resulting from scaffolds and equipment being too close to power or utility lines, environmental conditions such as wind, rain, and the presence of hazardous substances, inadequate fall protection, and collapses of scaffolds owing to overloading.

Table 1 shows an analysis of the industrial accidents in Korea from 2013 to 2017 [2]. Among the factors in industrial accidents in the construction industry, fatal accidents owing to temporary objects account for a significant proportion (approximately 22.8%). It well shows that a high probability of fatal accidents exists in construction site because various temporary equipment and structures are frequently used [3].

In addition, considering the accident status by type of scaffold shown in Table 2 [2], if accidents caused by mobile scaffolds are combined with accidents from using a ladder in a work environment requiring the use of the mobile scaffold, it accounts for a more significant proportion, i.e., 29%. As scaffolds are a temporary structure used in most construction sites, the safety accidents thereon have continued to directly contribute to the deaths and injuries of workers. As shown in Table 3 [2], the status of scaffolding accidents by type of occurrence, the most frequent fall occurred, followed by unbalanced motion, crushing, electric shock, collapse, and hit. As a result of analyzing the accident cases, it was caused by defects in the main member, the defect in the connecting member, and the buckling of the assembly. Therefore, it was identified that performance evaluation of the buckling behavior and load-bearing capacity of the assembly unit as well as the performance of the member was also necessary.

**Figure 1:**
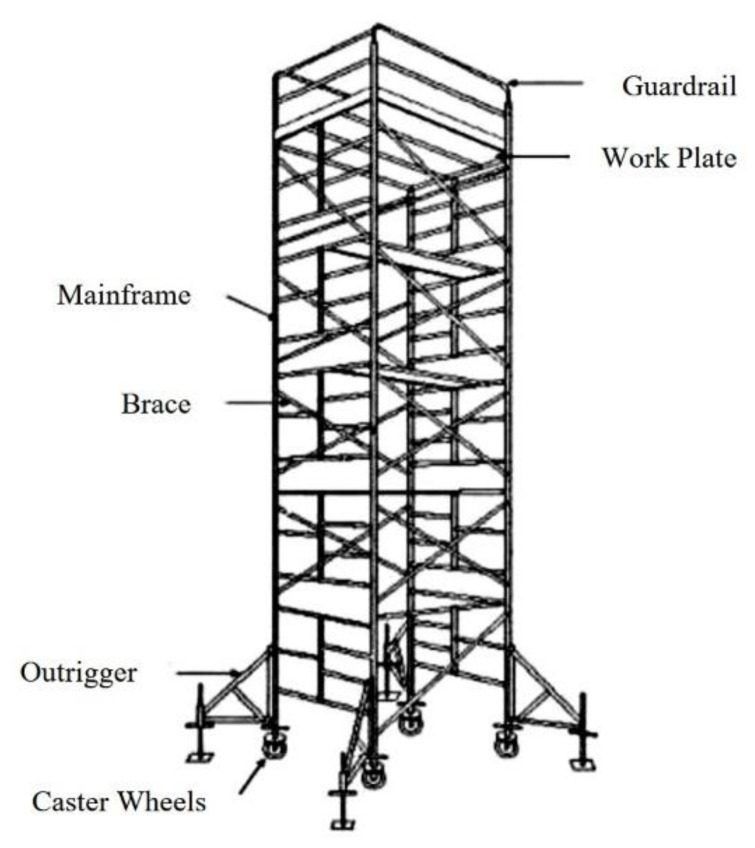
Components of mobile scaffold.

The mobile scaffold in Figure 1 is a scaffolding structure in which a work plate and safety handrail are installed on the top floor of a frame structure fabricated in the form of a tower, and in which caster wheels are attached to the bottom of each vertical member. These mobile scaffolds can not only be easily changed in height but can also be moved using the caster wheels. This type of scaffold is mainly used for finishing work, such as for interior ceilings and walls. Securing the stability of temporary structures for high-height work such as on scaffolds is the most important measure for preventing the frequent fall accidents at construction sites. In addition, such stability should be secured based on a safe and reasonable structural performance evaluation.

The structural performance of a mobile scaffold may depend on the structural characteristics of the assembly, rather than on the performance of a single member. In other words, if safety certification standards and performance evaluation methods are established in consideration of the actual usage, clear information on the structural performance can be provided to users before product use.

In Korea, there are safety certification standards applied for frequently used temporary equipment, such as scaffolds and shoring. However, the standards concern the strength criteria for the member itself, rather than the global load-carrying capacity. Therefore, it is difficult to review whether the fabricated mobile scaffold has sufficient load-carrying capacity, or to confirm the structural safety considering the various uncertainties affecting the structural performance.

Several countries have proposed certification standards and performance evaluation methods for mobile scaffolds. Like Korea, Japan implements a certification system. However, there is a separate approval system for fabricated mobile scaffolds, based on the structure-level certification system. In the case of the United States, performance standards and evaluation methods have been presented for prefabricated steel pipe frame scaffolds, but standards have not been provided for fabricated mobile scaffolds with caster wheels. In Europe, performance standards and evaluation methods have been presented for mobile scaffolds in which all members are combined; the strength and stiffness values of the structures are described in detail.

In this study, rational safety certification standards and evaluation methods are suggested for fabricated mobile scaffolds. The suggested safety certification standards present structure-level criteria for checking the load-carrying capacity, horizontal stiffness of the structure, and overturning risk. It is expected that the structural performance for safety can be directly checked based on the suggested safety certification standards and performance evaluation methods during the safety certification stage.

## 2. Literature Review

### 2.1. Study on the Structural Behaviors and Safety Certification Standards for Scaffolds

Structural analyses and experimental research are being conducted on assembled temporary equipment overseas. In general, the structural performance evaluation is performed on the entire structure, and extreme behavior analyses are performed depending on the presence or absence of members constituting the temporary equipment. In addition, studies are being conducted on the inelastic effect and geometric nonlinearity of the materials. However, few studies have been conducted on fabricated mobile scaffolds. Unlike other temporary equipment, specific safety certification standards and performance evaluation methods for mobile scaffolds with caster wheels have not been specifically presented except in Europe, and studies on the extreme behavior characteristics of such structures have not been conducted.

Chandrangsu (2009) [4] studied a rational structural modeling technique for evaluating the overall structural performance of the system unit of a scaffold. It was conducted based on the definition of appropriate finite elements and boundary conditions for simulation of the major structural members, connection conditions between members, and analysis units. The ultimate behavior analysis technique for structures was proposed through a nonlinear inelastic structural analysis considering both the material inelastic effect of steel and the geometrical nonlinear effect of the structure as a whole (or of individual member).

Peng et al. (2007) [5] analyzed the effects of the structural geometry and gradual load on the overall buckling strength of a fabricated scaffold. The overall buckling analysis was performed by directly implementing the horizontal and sloped members and end support conditions directly affecting the buckling strength of the compression and compression members (the main members of the scaffold) on the structural analysis model. As an analysis model, a three-story system scaffold was studied, and a nonlinear inelastic structural analysis was performed to consider the geometric nonlinear effect owing to the inelastic effect of steel and beam-column effect of the member.

Liu et al. (2010) [6] studied the structural stability of a steel pipe assembly type scaffold without braces. In general, for frame structures without braces, the main buckling mode is accompanied by a pronounced overall lateral deformation of the frame when compressive forces act on the vertical members. Therefore, the effective buckling length was long, and the buckling strength was also evaluated as very low. This structural behavior was analyzed through structural analyses and experiments.

Kim et al. (2021) [7] identified the structural behavioral characteristics of structures in units of finished products by conducting actual experiments and structural analysis studies on assembled mobile scaffolds distributed in Korea. The extreme behavior characteristics and load capacity of each structure were analyzed by conducting an experiment and structural analysis comprising loading a vertical load while using the material and height of the fabricated mobile scaffold with the caster wheels as the parameters. Thus, it was understood that the load capacity that the structure could withstand may vary depending on the height of the mobile scaffold and whether the caster wheels were attached, even if the performance standards of the members satisfied the safety certification standards for temporary equipment. Therefore, it was argued that it was necessary to establish reasonable safety certification standards and performance evaluation methods for the entire structural unit.

From analyzing the existing studies on the safety certification systems and performance evaluation methods for temporary equipment in Korea, it can be concluded that the existing safety performance standards for evaluating member units are difficult to use to clearly evaluate the structural behavior characteristics of an entire structure. To evaluate the structural performance of the assembled temporary equipment, it is suggested that not only the performance of the members, but also the connection conditions between the members, flexural stiffness ratio, load conditions, and presence or absence of installation of braces should be considered. Although studies on temporary equipment assembled in Korea are being actively conducted on system scaffolds and temporary shores, research on mobile scaffolds remains insufficient.

Bae and Lee (2002) [8] indicated that the distribution of untested products or products that have failed to pass performance tests was the cause of the continuous occurrence of industrial accidents, despite the implementation of the performance test system based on the Industrial Safety and Health Act of Korea. Therefore, they suggested the need for the consistent establishment of temporary equipment management standards and institutional improvements.

Lee and Choi (2013) [9] emphasized the current safety certification standards for temporary equipment in Korea are presented only to evaluate whether the individual components satisfy such performance standards. Accordingly, the possibility that the over-design of individual members may be induced was mentioned. Therefore, for a more reasonable safety certification system, it was proposed to introduce a safety certification system reflecting the structural characteristics of the fabricated temporary equipment and finished products.

Won et al. (2016) [10] compared domestic and foreign systems and standards related to temporary equipment, and suggested improvement directions for the safety certification system for temporary equipment in Korea. They proposed a separate safety certification committee for various types of temporary equipment, finished assemblies using new materials, and overseas temporary equipment.

Kim et al. (2017) [11] conducted a study on methods for securing the safety of temporary structures by securing the quality of the temporary materials. Accordingly, a stakeholder awareness analysis was performed on the safety certification labeling system. In addition, a plan to improve the safety certification mark was presented among the pending issues related to the safety certification mark.

Jeong et al. (2020) [12] conducted a feasibility study for the transition to a fabricated temporary equipment (system unit) safety certification system. Through a structural analysis, the overall structural behavioral characteristics of a system scaffold and temporary shore were analyzed. In addition, it was identified that the connection conditions and presence or absence of braces had dominant influences on the behavior of the temporary structure. Accordingly, it was argued that it was necessary to establish a performance evaluation and test method for assuring the safety and quality of temporary structures.

Bong et al. (2019) [13] studied the rotational stiffness and moment capacity of the wedge connection of the scaffold and Lee et al. (2020) [14] studied the effects of the installation detail on the ultimate behavior of the scaffolds. They well presented the load-carrying capacity of the scaffold is directly affected by many factors related to the details of the main components and their connections.

As shown in the above paragraphs, many researchers have concluded that the ultimate behavior and the load-carrying capacity of the scaffolds, the type of lattice or even frame structures, are determined by the details of the combination of main members. It means that the global strength of the assembled scaffold cannot be directly estimated by the strength of the member itself.

### 2.2. Safety Certification Standards and Performance Evaluation Methods for Temporary Equipment

#### 2.2.1. United States

In the United States, occupational safety and safety programs are implemented throughout the industry through the Occupational Safety and Health Administration under the Department of Labor, in accordance with the Occupational Safety and Health Act. Accordingly, the American National Standards Institute (ANSI), a non-profit private organization, plays a role in the general management and coordination of the various standards. The ANSI has developed, disseminated, and applied standards for various items [15]. In addition, it has provided consistent and continuous mutual protection by introducing a conformity assessment system. There is no single standard mark recognized at the national level in the United States. However, each certification body accredited by the ANSI operates a certification system to issue certificates and permit the use of certification marks.

Its agency, the Scaffold and Access Industry Association (SAIA), is responsible for the design, manufacturing, and performance testing of scaffolds and their components. The safety certification standards presented by SAIA do not include standards for the assembled mobile scaffold of the entire structure unit; this is intended to be presented in this study. However, the performance standards are presented for a fabricated scaffold without wheels, that is, a complete scaffold with welded frames. 

In the vertical load loading test of a scaffold, a strength test is performed by applying a load to four vertical members [15]. Test A presents a test method for a single-bay scaffold consisting of three stories, and Test B presents a test method performed in a multi-bay, that is, two or more scaffolds connected in the horizontal direction.

In addition, the performance standards and test method for the caster wheel are presented as a single-member test method [15]. The test methods for the torque test in the radial direction, torque test for the swivel brake, torque test for the wheel brake, and vertical load test are presented. However, as mentioned above, a test method for the entire structural unit, including the caster wheels, is not presented.

#### 2.2.2. Europe

Europe has proposed European standards for unifying the levels of standards among European Union countries. In Europe, the British Standards Institution, German Institute for Standardization, and French National Standards Agency are preparing regulations and detailed standards for scaffold installation, constituent members, materials, safety performance requirements, test methods, etc.

In the standard for mobile access and working towers made of prefabricated elements (EN 1004-1) [16], the materials, dimensions, design loads, safety, and performance requirements of the fabricated mobile scaffolds are presented. The European safety certification standards and performance evaluation methods for fabricated mobile scaffolds can be seen as the only standards presented for finished products (i.e., including caster wheels).

The European mobile scaffold design standards suggest a height limit for the structure according to the stiffness of the fabricated mobile scaffold [16]. These methods were used as a reference to prepare the safety certification standards and performance evaluation method for the fabricated mobile scaffold in this study.

#### 2.2.3. Japan

In Japan, the Japanese Temporary Industry Association manages a certification system for temporary equipment. The system for temporary equipment is divided into a certification system, approval system, single product approval system, and applied factory system. Among these systems, the certification system can be seen to have content similar to that of Korea’s temporary equipment certification system. The accreditation inspection system is operated under the legal basis of the Occupational Safety and Health Act (Articles 42 and 119 of the Act, Article 13 of the Enforcement Decree) [17]. The performance test items are classified into 49 standard products, 19 legally regulated items, and 30 performance test enforcement agencies’ standard items. The performance is checked using a sampling test. 

As mentioned above, the Japan Temporary Industry Association operates an approval system separately from the certification system. This system is not subject to the certification system for temporary equipment and is a system for confirming the safety of temporary structures as assembled with an entire structure. In other words, the performance certification system for temporary structures in Japan is based on the safety certification standards for single-member units, as in Korea. Accordingly, the structural performance of the temporary structure of the entire system unit is not subject to certification. Therefore, an approval system is being used to evaluate the structural performances of new materials and newly assembled temporary structures. In other words, it is an approval procedure that incorporates assembly and usage methods by conducting a system-level strength test. 

This approach is similar to the safety certification standards for the fabricated mobile scaffold of the entire product unit, as proposed in this study. However, Japan’s approval system for temporary structures is not mandatory owing to legal force. However, in response to a request for a structural performance evaluation of an assembled temporary structure, the manufacturer can obtain voluntary approval. Therefore, there is no performance standard for approval, and the Japan Temporary Industry Association’s test method is being used for performance evaluations.

#### 2.2.4. Korea

Korea examines manufacturers’ technical capabilities, production systems, and product performance for temporary equipment classified according to Article 34 of the Occupational Safety and Health Act (an act on safety certification), and Article 36 of the Industrial Safety and Health Act (an Act on voluntary safety verification and reporting). In addition, a safety certification mark can be used if it meets the safety certification standards.

As shown in Table 4, temporary structures and equipment are divided into 12 types and 33 items subject to mandatory safety certification, and eight types subject to voluntary safety reports. Among them, mobile scaffold members are classified as subject to mandatory safety certification, and performance standards and test methods for each member of the mobile scaffold are specified in accordance with the mandatory safety certification notice for protective devices. As described above, the safety certification standards for mobile scaffolds in Korea exist only for each member, and not for the entire structure. A performance evaluation method is also suggested for conducting tests, but only on the single members of the system scaffold, temporary shore, prefabricated scaffold, and mobile scaffold.

The Ministry of Employment and Labor Notice No. 2018-54, on the procedures for safety certifications and autonomous safety confirmation reports, presents performance standards and test methods for mobile scaffold members [18]. The members constituting the mobile scaffold are divided into the mainframe, caster wheel, handrail, and outrigger. Accordingly, material standards are suggested for each member, but it is also suggested that general structural steel pipes be used for the materials of most members, except for the caster wheels. The structural and material standards for each member are shown in Table 5 and Table 6, and the test performance standards for the mainframe are shown in Table 7.

Similar to the Japanese certification system, the safety certification system for temporary equipment in Korea proposes to conduct verification for each member unit. However, the US and Europe are proposing safety certification standards for temporary structures in finished products, and are proposing broader and more flexible standards for materials and structural shapes. Insofar as Europe is concerned, design standards, safety certification standards, and performance evaluation methods for mobile scaffolds fabricated with caster wheels have been presented.

### 2.3. Design Standards for Mobile Scaffolds in Korea

In Korea, a mobile scaffold is manufactured according to the design standards for scaffolds and safety facilities. The design standards are divided into general matters, materials, and designs. In the general section, the scopes of the scaffold, design load, and structural design are presented, and in the materials section, material standards for each scaffold are presented. In the design section, standards for the applied safety factors and considerations are presented for the design of scaffolds and safety facilities. 

The safety certification standards and performance evaluation methods for the fabricated mobile scaffold presented in this study were prepared based on the design standards for scaffold and safety facilities in Korea. The main contents of the cited items are as follows.

#### 2.3.1. Vertical Load

The vertical loads of a scaffold include dead loads and the working loads of the scaffold and work plate. Among them, the weight of the working platform, a dead load value, should be 0.2 kN/m^2^ or more. The working load includes the workers, materials, and tools used by the workers and is applied separately, as shown in Table 8.

#### 2.3.2. Horizontal Load

As for the horizontal load acting on the scaffold, the larger wind load and a horizontal load corresponding to 5% of the vertical load act on the member. However, the fabricated mobile scaffold proposed in this study is mainly used indoors. It has also been proposed to bind the scaffold to a fixed support, such as a wall, during work to reduce the effect of the horizontal loads. Therefore, it was assumed that the applied horizontal load (corresponding to 5% of the vertical load) was larger than the wind load.

#### 2.3.3. Materials

The materials for scaffolds and safety facilities are generally divided into steel pipes for single-pipe scaffolds, clamps, and work scaffolds. In particular, a mobile scaffold must comply with the safety certification standards for protective devices or KSF 8011 (members of a mobile steel pipe scaffold). In addition, the allowable tensile performance of each member follows the allowable tension stress. The safety certification standards for the mainframe are listed in Table 7.

#### 2.3.4. Structural Design

In the case of the mobile scaffold, the connection conditions for each member are applied as shown in Table 9. In principle, the boundary condition of the scaffold supports is regarded as a hinge.

#### 2.3.5. Safety Factor

For the safety factors of the members used in scaffold and safety facilities, the values shown in Table 10 are applied, based on performance test values from an authorized testing laboratory.

#### 2.3.6. Comparative Analysis of Standards Related to Mobile Scaffold

Korea does not present a separate design standard for the mobile scaffolds. It is integrated and presented in the scaffold and safety facility design standards. Therefore, specific standards for mobile scaffold are presented in the safety certification standards. In the case of Europe, separate design standards for prefabricated mobile scaffolds are presented. Therefore, the main criteria for mobile scaffolds were classified as shown in Table 11, and the performance standards for mobile scaffolds in Korea and Europe were compared and analyzed.

## 3. Study on Rationality of the Current Safety Certification Standards for Assembled Mobile Scaffolding through Structural Analysis

### 3.1. Performance Evaluation Method for the Mainframe of Mobile Scaffold

**Figure 2:**
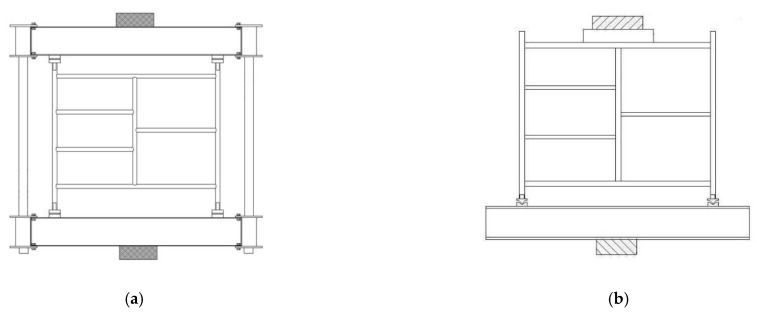
Performance evaluation test method of mobile scaffold mainframe. (**a**) Compression test of mainframe. (**b**) Deflection test of mainframe.

The mainframe of the mobile scaffold is the most important member to the structural performance of the structure. In accordance with the Korean Occupational Safety and Health Act, the Ministry of Employment and Labor’s Safety Certification Notice for Protection Devices (Ministry of Employment and Labor Notice No. 2021-22) has presented the performance standards and test methods for mobile scaffold members, as shown in Figure 2.

Therefore, in this study, a structural analysis was conducted to understand the ultimate behavior characteristics of the mainframe and finished product, where the vertical load acting on the mobile scaffold was considered as the largest. Through structural analysis, the ultimate loads of the fabricated mobile scaffold mainframe and those for the single-, two-, and three-story versions of the finished product were compared. This was to analyze the rationality of the existing safety certification standards for limiting the ultimate load and vertical displacement of a mainframe. In addition, when a vertical load was applied to an actual finished product, it was intended to determine whether the performance standard of the mainframe presented in the safety certification standard was satisfied.

### 3.2. Specifications and Structural Analysis Method

Table 12 shows the specifications for the mainframe of the fabricated mobile scaffold used in the experiment and the single-, two-, and three-story versions of the finished product. As for the fabricated mobile scaffold, the products currently the most sold in Korea were selected.

In accordance with the method suggested in the test method for mobile scaffold members, the critical buckling load was measured by applying a load to the top of the two vertical members while preventing the side sway of the mainframe. In the case of the single-, two-, and three-story versions of the fabricated mobile scaffold in the state of a finished product, the ultimate load was calculated by applying a vertical load to the top of the four vertical members. However, to accurately compare the performance evaluation results between the mainframe and finished product for the vertical load, a structural analysis was performed on the finished product without the caster wheels.

**Figure 3:**
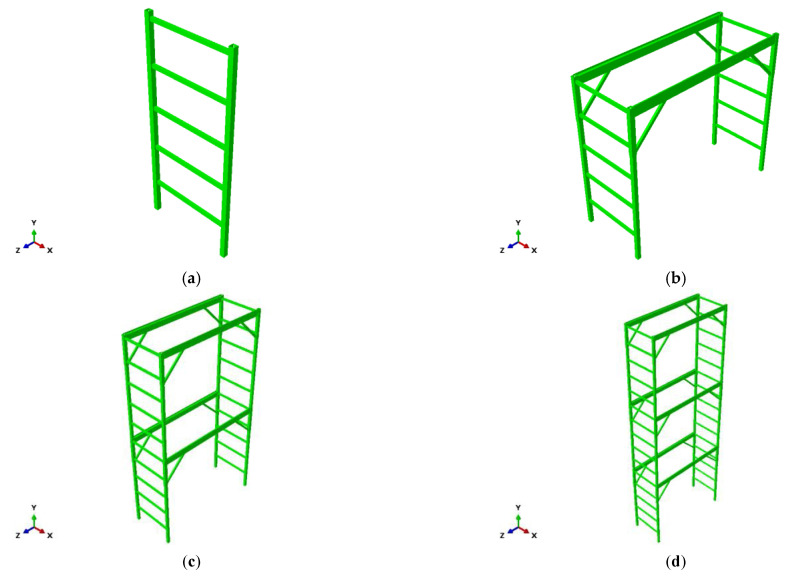
Structural analysis models of fabricated mobile scaffolds and mainframe. (**a**) Mainframe. (**b**) Single-story. (**c**) Two-story. (**d**) Three-story.

In particular, a finite element analysis was conducted using ABAQUS V2020. The numerical models included the vertical and horizontal members, as shown in Figure 3. All line members were modeled using a three-dimensional nonlinear beam element [19,20,21]. To investigate the ultimate behaviors of the mainframe model and fabricated mobile scaffolds, a nonlinear inelastic analysis was conducted. For the nonlinear inelastic analysis, the arc-length method was adopted as the incremental iterative analysis scheme [22]. 

The boundary conditions of the structural analysis model are shown in Table 13. The connection boundary conditions were constrained in all the x, y, z, xθ, yθ, and zθ directions, and the support boundary conditions were constrained only in the x, y, and z directions.

### 3.3. Structural Analysis Results

**Figure 4:**
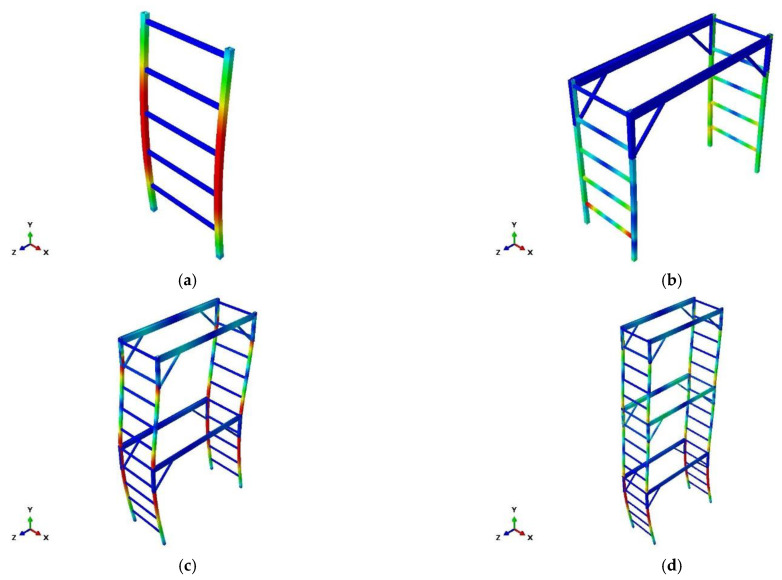
Deformation shapes of fabricated mobile scaffolds and mainframe according to structural analysis. (**a**) Mainframe. (**b**) Single-story. (**c**) Two-story. (**d**) Three-story.

Figure 4 shows the deformed shapes and load–displacement curves of the models as obtained using the nonlinear inelastic analysis. Although the magnitudes of the ultimate loads acting on each structural analysis model are different, in all structures, the vertical member is the most important member for resisting the vertical load. 

As shown in Table 6, the standards for the compressive load of the mainframe of the mobile scaffold specified in the safety certification standards and performance evaluation method require a performance of 44,000 N or more. In addition, the vertical deflection is limited to 10 mm or less. Therefore, in the case of the finished product, because two mainframes are included, the required performance of compressive strength for the structure is 88,000 N or more.

**Figure 5:**
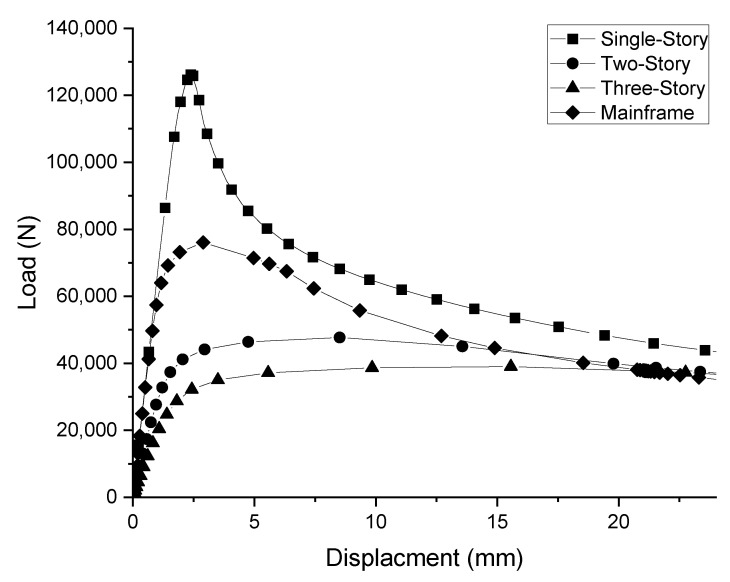
Load–displacement relationship of the fabricated mobile scaffolds and mainframe.

The structural analysis results in Figure 5 show that the ultimate load acting on the mainframe is 38,037 N. Therefore, assuming a finished product including two mainframes, the ultimate load is 76,076 N. This result falls short of the safety certification standards for the mobile scaffold mainframe. This is because the material and structural specifications of the fabricated mobile scaffold mainframe do not meet the scaffold design standards. In the case of the single-story fabricated mobile scaffold, the ultimate load reaches 126,205 N, greatly exceeding the safety certification standard for the mainframe. It is judged that the ultimate load is large because the vertical load acts under the connection and constraint conditions bound by the horizontal members and braces, etc. constituting the mobile scaffold in which the two mainframes are assembled. However, as the number of stories (that is, the height of the fabricated mobile scaffold) increases, the magnitude of the ultimate load is significantly reduced. In the case of the two-story fabricated mobile scaffold, the ultimate load is 47,693 N. Moreover, in the case of the fabricated mobile scaffold with three stages, the ultimate load is 39,005 N. As the height of the structure increases, the buckling length for the vertical loads increases. Accordingly, buckling of the vertical member can occur even from the action of a small vertical load.

As a result of the structural analysis of the fabricated mobile scaffold mainframe and finished products, it can be seen that the change in the ultimate load according to the changes in the assembly conditions and height is large. In the case of the fabricated mobile scaffold, the ultimate load is below the safety certification standard for a mainframe with that material and structure, and does not meet the design standard for the scaffold. In addition, the ultimate load generated in the single-story assembly far exceeds the safety certification standard for the mainframe. However, as the number of stories of the assembly increase, the buckling length for the vertical load increases, resulting in a sharp drop in the ultimate load.

Therefore, it is difficult to secure the safety of a structure by simply using member-centered safety certification standards and performance evaluation methods. In particular, to evaluate the ultimate load for a structure in an assembled state, it is necessary to prepare a safety certification standard for the finished product, rather than individual safety certification standards for the member unit. Thus, it is necessary to secure the safety of workers by presenting reasonable safety certification standards suitable for various materials and structures.

## 4. Suggestion of Safety Certification Standards for Fabricated Mobile Scaffolds

The safety certification standards for fabricated mobile scaffolds must comply with the following principles. First, in a fully assembled state, it must be possible to intuitively check and verify the structural performance, and the unfavorable constraints from the existing safety certification standards must be deleted. In addition, increased efficiency can be pursued by simplifying the authentication process for each member unit, i.e., for the entire structure unit authentication process.

In a general certification standard, the contents of the material and structural standards are presented. In addition, performance standards and evaluation methods are presented for the vertical load, horizontal load, and overturning safety. The method of calculating the load according to the load class, or the method of determining the horizontal load, should not deviate from the framework of Korea’s design standards for scaffold and safety facilities. Therefore, in this study, updated standards were derived, such as those for the vertical load, horizontal load, and safety factors suggested in the design certification.

### 4.1. General Safety Certification Standards for Materials and Structures

The scope of application for the safety certification standards for fabricated mobile scaffolds was set as certification standards for materials and structural types different from those comprising the general mobile scaffolds manufactured in units of members in Korea. The manufacture was required to follow the assembly or installation method provided by the manufacturer. As shown in Table 14, the material was made to include a square tube and aluminum tube in addition to the steel tube, i.e., the main material of the general mobile scaffold.

**Figure 6:**
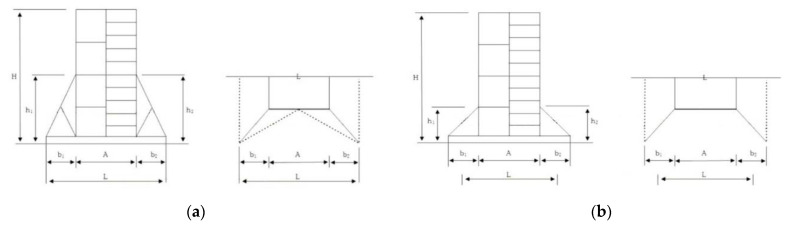
Height limitation of fabricated mobile scaffold. (**a**) Method A. (**b**) Method B.

The width of the structure was suggested to have a value greater than or equal to the minimum size of the working scaffold according to the Korean working scaffold and passage design standards. In addition, the height of the structure was based on the height limit of the scaffold specified in the guidelines for the use of mobile scaffolds manufactured by the Korea Occupational Safety and Health Agency [23]. As shown in Figure 6 and Equation (1), a height limit was suggested for the mobile scaffold, and a height for a fabricated mobile scaffold was also suggested according to this guideline.

(1) H≤7.7L−5.0

Here, H: height from the bottom of the caster wheel to the work plate (m); and L: spacing of the main axis (short side) of the caster wheels (m).

### 4.2. Safety Certification Standards for Strength Safety of the Fabricated Mobile Scaffolds

Equation (2) is suggested for the vertical load that dominates the strength of the structure. $w_{1}$ is the vertical load per unit area for each work class presented in the design standards for scaffold and safety facilities in Korea. The work classes are divided into three classes: light work, heavy work, and heavy material work. The unit weights for each class are shown in Table 7.

(2) $$P_{v}=3\times \left(w_{1}+w_{2}\right)\times A\times n$$

In the above, P: maximum load for each story of work plate (kN); $w_{1}$: vertical load per unit area by work grade ($\mathrm{kN}/m^{2}$); $w_{2}$: unit load of work plate ($\mathrm{kN}/m^{2}$); A: work plate area ($m^{2}$); n: number of story of work plate; and a compression safety factor of 3 is applied.

*W*_2_ is the unit weight of the work plates, and was obtained by reference to Korean work plates and passage design standards. 

The height of the first stage of the assembled mobile scaffold was approximately 2 m, and most instances of the fabricated mobile scaffold comprised single-, two-, and three-story examples. That is, the maximum height of the fabricated mobile scaffold was limited to approximately 6 m. Considering this, the n value was presented such that the three types of loads could be calculated. In addition, in the design standards for scaffold and safety facilities in Korea, the safety factor for the compression of structures is presented as 3.0, and this was applied to the proposed Equation (2). It was considered that as the load acts on all installed working scaffolds, as the height of the structure increases, that is, as the number of working scaffolds increases, the loading load acting on the structure increases proportionally.

Table 15 shows an example of a loading load classification from applying the proposed Equation (2), based on the width of the working scaffold of a general mobile scaffold. The general mobile scaffold’s work plate is the maximum value of the area suggested in the safety certification standards for the fabricated mobile scaffold. Therefore, the vertical load per unit area of the fabricated mobile scaffold is the maximum value, as shown in Table 15. The example is an example calculated by the area (1000 mm × 1850 mm) of the work plate of a general mobile scaffold.

### 4.3. Safety Certification Standard for Limiting Horizontal Displacement of Fabricated Mobile Scaffold

As the purpose of using the mobile scaffold is often to work in a space with a narrow area and high height, the area of the work plate has a structure in which the height is gradually increased in a fixed state. Therefore, the rigidity of the structure against a horizontal load has an important influence on the working environment.

As mentioned above, the design standards for scaffold and safety facilities in Korea suggest that the larger value between the wind load and a load corresponding to 5% of the vertical load is applied as the horizontal load. Among temporary structures, a mobile scaffold is mainly used indoors rather than in outdoor worksites, and it has been suggested to bind such scaffolds to fixed structures such as walls during the work. Therefore, it is reasonable to apply 5% of the vertical load as the horizontal load rather than the wind load.

The standards for horizontal displacement according to the height of a mobile scaffold are not separately presented in the design standards for scaffold and safety facilities in Korea. Therefore, Equation (3) was proposed by borrowing the horizontal displacement limit value according to the height of the structure as suggested in the European design standard for mobile scaffolds (EN 1004-1).

(3) $$d_{h}=\frac{1}{30}h_{max}$$

here, $d_{h}$: maximum horizontal displacement (m); and $h_{max}$: maximum height of fabricated mobile scaffold (m).

For example, if the maximum height of the scaffold is 6 m, the horizontal displacement of the uppermost work plate should not exceed 0.2 m when the horizontal loads are calculated for each work class action. Table 16 shows the horizontal load values derived based on the load classification in Table 15 for each work class of the general mobile scaffold.

### 4.4. Safety Certification Criteria for Overturning Safety of Fabricated Mobile Scaffold

The safety certification standards regarding the overturning safety limit the lifting of the feet attached to the bottom of the fabricated mobile scaffold when an eccentric load is applied, e.g., from a worker’s movement or from loading on the top work plate of the scaffold.

In Equation (4), 0.9 is the overturning moment that one worker can generate at the top of the fabricated mobile scaffold. This value considers the weight of the worker (100 kg) and the location of the worker who may be the most vulnerable to overturning. This equation considers the overturning safety factor of 3.0 suggested in the design standards for scaffold and safety facilities in Korea.

(4) $$P_{h}=\frac{0.9}{h_{max}}\times 3$$

In the above, $P_{h}$: horizontal load for overturning safety (kN); $h_{max}$: maximum height of fabricated mobile scaffold (m); and an overturning safety factor of 3 is applied.

For example, if the height of the fabricated mobile scaffold is 6 m, the maximum horizontal load to secure overturning safety is 0.45 kN. Therefore, when this horizontal load acts on the structure, there should be no lifting of the caster wheels.

## 5. Performance Evaluation Method for Fabricated Mobile Scaffolds

### 5.1. Performance Evaluation Method for Strength Safety of the Fabricated Mobile Scaffold

**Figure 7:**
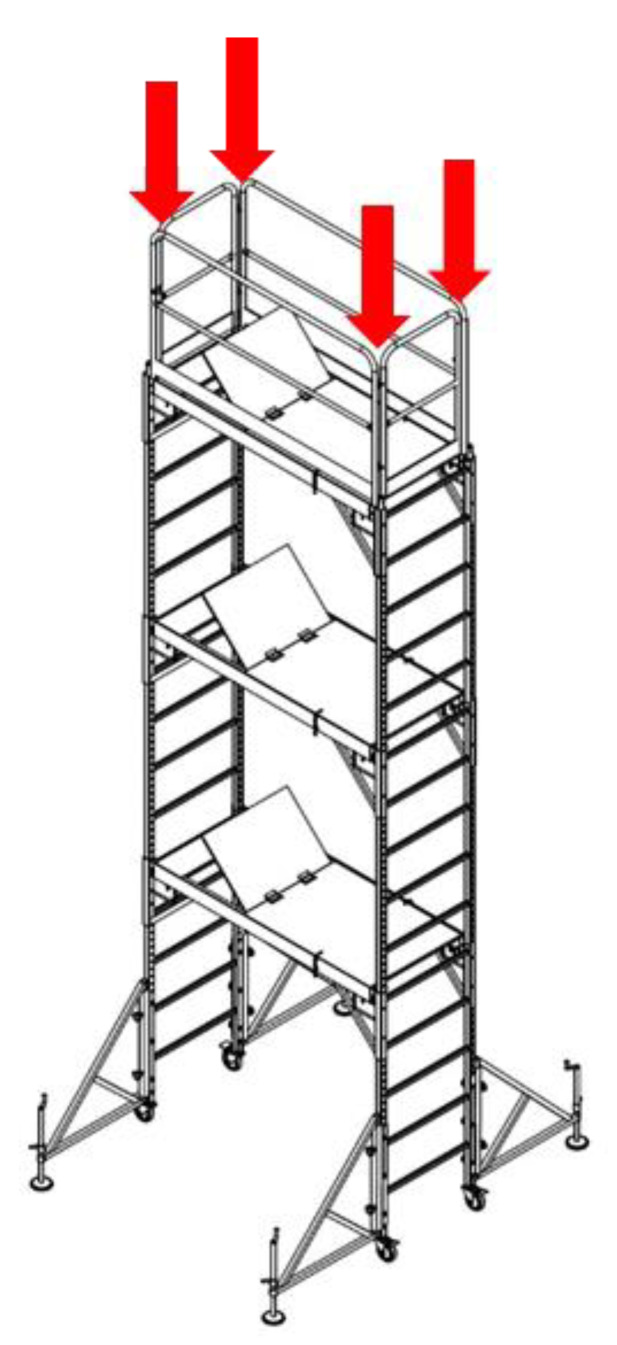
Vertical load test method.

As shown in Figure 7, the strength performance test for the vertical load acting on the fabricated mobile scaffold measures the maximum load that the structure can withstand by applying the same compressive force to the four vertical members bearing the vertical load. The resultant value from the test is compared with the calculated value according to the proposed equation presented in the safety certification standard for strength safety to evaluate the performance class.

In this test, the specimen must be performed in a fully fabricated state, and the number of stages is divided by the height of the work plate. This approach is prescribed for accurate fabrication based on the assembly drawings provided by the manufacturer. In addition, any artificial adjustment of the members or connections between the members is restricted.

To apply an even vertical load to the four vertical members, the test can be performed after removing the toe plate and handrail, as these may be a hindrance to the experiment. In addition, the load can be applied after binding the iron support to the uppermost part of the vertical member, so that it tightly adheres to the jig. The height-adjustable caster wheels can be extended to the maximum to realize the adverse geometrical conditions.

### 5.2. Performance Evaluation Method for Limiting Horizontal Displacement of Fabricated Mobile Scaffold

The stiffness test of the fabricated mobile scaffold measures the horizontal displacement generated by applying an equal horizontal load to two vertical members located on one side of the uppermost part of the structure. During the test, a sufficient ballast can be installed at the bottom to prevent the structure from overturning. In addition, the caster wheel located on the surface not undergoing a horizontal load can be fixed so that it does not move.

**Figure 8:**
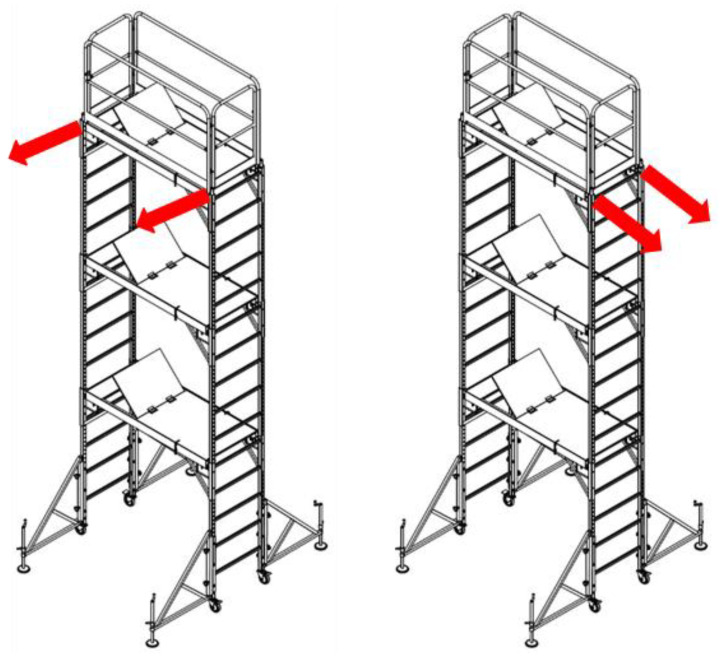
Horizontal load test method.

As shown in Figure 8, the test load in this study comprised two horizontal loads applied perpendicular to the outer surface of the scaffold. Therefore, it was suggested that the test be repeated at 90° to the first side after applying a horizontal load on one side.

### 5.3. Performance Evaluation Method for Overturning Safety of Fabricated Mobile Scaffold

The location and method for applying a horizontal load in the performance test for overturning safety are the same as those of the performance test for limiting the horizontal displacement. However, in this study, a fully fabricated mobile scaffold equipped with outriggers was used as the test object. In addition, unlike the experiment for limiting the horizontal displacement, the experiment was performed with the ballast removed.

In the same way as the stiffness test, it was repeatedly performed at 90° of the first horizontal load bearing surface, and it was suggested to check whether there was a lifting of the caster wheel. In addition, during the experiment, the caster wheels were fixed in the most unfavorable direction for overturning the structure.

## 6. Application Example of the Suggested Safety Certification Standards and Experimental Evaluation for the Fabricated Mobile Scaffold

**Figure 9:**
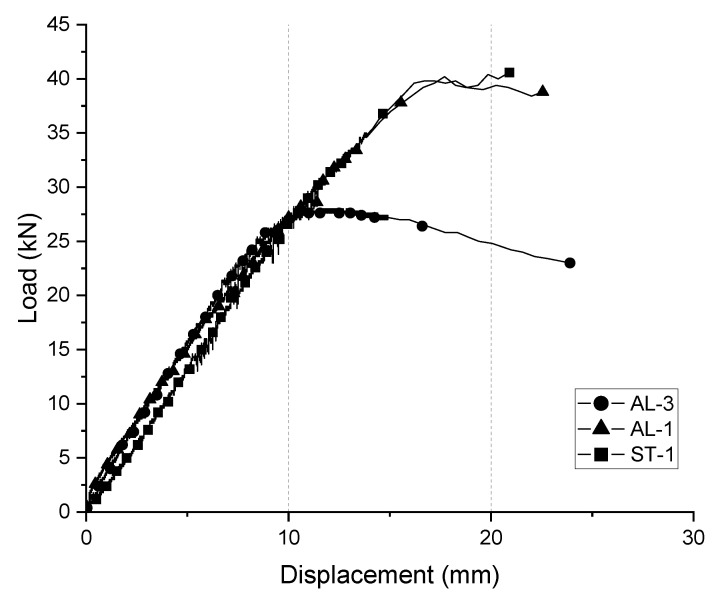
Load–vertical displacement curves of specimens.

Based on the safety certification standards for strength safety presented in this study, the work classes were classified for the three products for which the vertical load tests were performed. As shown in Table 17, two 1-story and one 3-story fabricated mobile scaffolds were examined. Also, one of the 1-story specimens was fabricated with aluminum and the other was fabricated with steel. Details of the experiments were presented in a previous study (Kim et al., 2021) [7]. Figure 9 shows the load–displacement curves for the vertical load applied to the three specimens. As can be seen from the experimental results, the experimental results for the single-story specimens show results similar to those of the steel and aluminum specimens. However, the loading capacity sharply decreases in the experimental results of the three-story specimen with a high height. According to the research, the governing factor on the ultimate behavior of the 3-story specimen was buckling of the vertical members rather than a failure of the caster wheel. Additionally, in the case of the single-story specimens, the destruction of the caster wheel occurs before the buckling of the vertical member. The experimental study clearly shows that the load-carrying capacity of the fabricated mobile scaffolds cannot be easily estimated by the member strength because the ultimate modes and the governing factor on the global strength are determined by the details of the combination of the main components as well as the material strength. Therefore, it is reasonable to judge a fabricated mobile scaffolding based on safety certification criteria for the overall shape of the structure, rather than using individual member performance-based safety certification criteria.

Table 18 shows the calculation of the suggested safety certification standards for the strength safety, based on the specifications of the specimen. 

Table 19 shows the classification of the work classes according to the experimental results. According to the value of the vertical load, the single-story aluminum scaffold was classified as a product with a work class of “3”, that is, a product capable of heavy work with heavy construction materials. However, the three-story fabricated mobile aluminum scaffold was classified as a product only suitable for heavy work, with a work class of “2”. According to the value of the vertical load, a single-story mobile scaffold fabricated with steel was classified as work class “3”, that is, a product capable of heavy work with construction materials.

A horizontal load test for limiting the horizontal displacement was not performed owing to the experimental conditions, but the horizontal load was derived by applying the safety certification standards for the horizontal displacement limit proposed above, according to the specifications of the specimens as shown in Table 20. When proposed horizontal load is applied according to each class and height, the specimen should be displaced less than the horizontal displacement value derived using Equation (4). In addition, the safety certification standards for overturning safety must be satisfied to fully satisfy the safety requirements for the structure.

From analyzing the load class classifications reflecting the experimental results, it can be seen that the load class that the structure can handle varies depending on the number of stories in the assembly, even if the performance meets the safety certification standards centered on the member units.

It is necessary to understand the structural behavior characteristics of the entire structure, which cannot be confirmed in the existing safety certification standards and performance evaluation methods, and to analyze the strength, stiffness, and fall safety of the structure according to various load conditions. Therefore, it is necessary to apply the new safety certification standards to the entire unit of the structure to calculate the appropriate work class(es) as the area of the work plate and number of stories changes.

## 7. Conclusions

In this study, rational safety certification standards and evaluation procedures are suggested for fabricated mobile scaffolds. The newly suggested standard includes global structural performance criteria related to the load-carrying capacity, horizontal stiffness, and overturning prevention, based on load tests for the assembled structures. By using the suggested standards and evaluation methods, the structural safety of mobile scaffolds assembled on-site can be directly reviewed during the safety certification stage.

1. The safety certification standards and performance evaluation methods for mobile scaffolds in Korea are presented, with a focus on the performance tests of each member constituting the scaffold. In Japan, like Korea, a member-based certification system is being operated, but a separate approval system has been prepared to perform global performance tests on temporary equipment in the assembly unit. In addition, although there is no certification system for temporary equipment in the US and Europe, performance standards and evaluation methods are presented in the design standards for fabricated scaffolds and fabricated mobile scaffolds. European design standards suggest performance standards for a complete mobile scaffold with attached wheels.
2. As shown in the analytical investigation of the load-carrying capacities of the assembled scaffolds, the structural strength and stiffness can be determined based on the details of the assembly. Therefore, the member-based safety certification criteria are not appropriate for fabricated mobile scaffolds used on-site with various assembly details, boundaries, and load cases.
3. To overcome the limitations of the member-based certification criteria, global performance-based safety certification criteria are suggested. The suggested standards include criteria reflecting the load-carrying capacity for vertical loads (for strength), horizontal stiffness (for serviceability), and overturning prevention.
4. Three work classes are suggested, considering the structural design standards. The criteria are determined based on the work plate area and height of the assembled structure. Therefore, safety certifications can be applied and examined while reflecting the details of the equipment usage on-site.

## Figures and Tables

**Table 1:** Fatalities related to scaffold work in Korea for 5 years (2013–2017).

Division	Construction Industry	Scaffold Work
Total	2134	488
Share	100%	22.8%

**Table 2:** Accident status by type of scaffold.

Total	Steel Pipe Scaffold	Ladder	Hanging Scaffold	Mobile Scaffold	Gang Form	Step Ladder	System Scaffold	Steel Pipe Support	Prefabricated Scaffold	Other
488 (100%)	213 (43.7%)	97 (19.9%)	63 (12.9%)	45 (9.2%)	37 (7.6%)	17 (3.5%)	9 (1.8%)	3 (0.6%)	2 (0.4%)	2 (0.4%)

**Table 3:** Accident status by type of occurrence.

Total	Falling	Unbalanced Motion	Underlay	Electric Shock	Collapse	Hit	Bump	Jammed	Overturning	Other
488 (100%)	417 (85.5%)	21 (4.3%)	13 (2.7%)	9 (1.8%)	8 (1.7%)	7 (1.4%)	6 (1.2%)	4 (0.8%)	1 (0.2%)	2 (0.4%)

**Table 4:** Subjects for safety certification and autonomous safety verification.

Mandatory Safety Certification Target	Voluntary Safety Report Target
1. A member for pipe support and support	1. Shelf Post
2. Prefabricated scaffold members	2. Steel pipe for single pipe scaffold
3. Mobile scaffold member	3. Fixed support hardware
4. Support hardware (except fixed type)	4. Hanging scaffold and absence
5. Fastener	5. Protective shelf
6. Prefab safety railing	6. Handrails for elevator openings
7. Fall or fall protection nets	7. Bracket for side wall
8. Sub-materials like or in combination with the provisions of items 1 to 7, etc.	

**Table 5:** Material performance standards for mobile scaffold.

Member	Material Performance Standards for Mobile Scaffold
Mainframe	(1) The material of the pillar member, lateral member and reinforcing member must be STK400 of KS D 3566 (carbon steel pipe for general structural use) or have mechanical properties equivalent to or higher than that of KS D 3566. (2) Each part of the mainframe must be free from significant damage, deformation, corrosion, or wear.
Caster wheel	(1) Main shaft and axle: SS400 of KS D 3503 (rolled steel for general structure) (2) Tire: KS B 6415 (industrial wheel) type 1 (limited to those specified in 6.2). It should be used with mechanical properties equivalent to or higher than this.
Handrail	(1) Footrest plate, post material, handrail material, brace material: STK400 specified in KS D 3566 (carbon steel pipe for general structure) (2) Bolts, nuts, pins, etc. among hardware for installation: SS330 specified in KS D 3503 (rolled steel for general structure) (3) Other parts of the installation hardware: SPHC specified in KS D 3501 (Hot-rolled mild steel plate and steel strip) must have mechanical properties equivalent to or higher than that of SPHC.
Outrigger	(1) Items for outriggers are in accordance with the standards set by the Korean Industrial Standard (KS F 8011).

**Table 6:** Structural performance standards for mobile scaffold.

Member	Structural Performance Standards for Mobile Scaffold
Mainframe	(1) The length between the centers of both vertical members should be 1200 mm or more and 1600 mm or less (2) The length of the vertical member must be 2000 mm or less. (3) The outer diameter of the vertical and horizontal member must be 42.4 mm or more. (4) In case of having an insertion tube at the upper end of the vertical member, the length of the part where the vertical member is inserted must be 95 mm or more, and it must have a structure that does not come off. (5) If there is no insertion tube at the upper end of the vertical member, it shall have a structure that does not break off by inserting a connecting joint that meets the provisions of Nos. 18 through 20 (6) The length of the horizontal reinforcing member used as a stepping platform and the part of the horizontal member shall be 300 mm or more, and the center distance between the horizontal stiffeners shall be 400 mm or less.
Caster wheel	(1) Among the main shafts, the length of the part inserted into the vertical member of the mainframe shall be at least 200 mm (95 mm in the case of the main axis having a separation prevention function). (2) The outer diameter of the wheel tire must be at least 125 mm. (3) The axle should be able to rotate around the main shaft.
Handrail	(1) The outer diameter of the posts, handrails and braces must be at least 21.4 mm. (2) When the handrail frame is installed on the mainframe, the height of the handrail frame must be at least 900 mm from the top of the workbench to the top of the upper handrail. (3) Intermediate handrails or braces must be installed in the middle of the handrail frame, and the intermediate handrail must be in the middle between the top of the workbench and the top of the upper handrail. (4) Installation hardware must have a strong structure that does not fall off easily during use. (5) The vertical member of the handrail must be of a structure that is inserted into the post of the handrail or that is connected by a connecting pin.
Outrigger	(1) Items for outriggers are in accordance with the standards set by the Korean Industrial Standard (KS F 8011).

**Table 7:** Test performance standard for mobile scaffold mainframe.

Items	Test Performance Standards
Compressive strength	44,000 N or more
Vertical deflection	10.0 mm or less

**Table 8:** Working load class according to the working classification of scaffold.

Work Class	Working Classification	Working Load
1	Light work requiring only light tools and scaffold to act as a passageway	$1.25\;\mathrm{kN}/m^{2}$ or more
2	Heavy work requiring the loading of construction materials	$2.5\;\mathrm{kN}/m^{2}$ or more
3	Material-heavy work, such as stone laying	$3.5\;\mathrm{kN}/m^{2}$ or more

**Table 9:** Connection conditions for mobile scaffold members.

Connection	Condition
Connection of vertical member and vertical member	Continuous condition
Connection of vertical and horizontal members	Continuous condition
Connection between mainframe and cross brace	Hinge connection

**Table 10:** Safety factor of scaffold and safety facility members.

Items	Safety Factor
Tensile	2
Bending	2
Shear	3
Compression	3
Overturning	2

**Table 11:** Comparison of standards related to mobile scaffolding in Korea and Europe.

	KOREA		EUROPE
	Safety Certification Standard for Mobile Scaffold Member	Design Standards for Scaffolding and Safety Facilities	Mobile Access and Working Towers Made of Prefabricated Elements
Material	- Steel only (except for the caster wheels) - Use STK400 of KS D 3566 (carbon steel pipe for general structure) for mainframe, guardrail and outrigger	-	- Steel, cast iron, aluminum alloys, timber - BS EN 12811-2 temporary works equipment–information on materials
Structural	- The length between the centers of both vertical member of the mobile scaffold is 1200 mm or more and 1600 mm or less. - The length of the vertical member is 2000 mm or less	-	- Suggestion of work plate size and structure height for whole structure - Present detailed specifications for each member
Load	- Compressive strength performance standard of the main frame: 44,000 N or more - Compressive strength performance standard of the caster wheel: 16,000 N or more	- Suggestion of vertical load per unit area for work classes - Consider vertical load, wind load, horizontal load and special load - The larger of the wind load and the horizontal load corresponding to 5% of the vertical load acts on the member. - Horizontal loads act independently in each direction and do not overlap. - Application of wind load does not consider the effect of working load.	- Suggestion of vertical load per unit area for work classes - Consider vertical load, wind load, horizontal load - Present service loads for the entire structure by group
Safety Factor	-	- The safety factor is applied based on the performance test value - Tensile 2.0, Bending 2.0, Shear 3.0, Compression 3.0, Overturning 2.0	- All permanent and variable loads: 1.5, accidental loads: 1.0 (Ultimate limit state) - Overturning: 1.5 or more
Performance Test	- In relation to Article 36 of the Act, the performance standards and test methods - Suggested standards for main frame, caster wheel, guardrail, and outrigger - Suggestion of performance evaluation method of each member - Absence of safety certification standards and performance evaluation methods for mobile scaffold in assembly	-	- Stiffness test on complete tower structure - Horizontal load 500 N: structure height 6 m, horizontal displacement 200 mm - The linear formula is not precisely correct but over the range of towers that this document covers.

**Table 12:** Specification of fabricated mobile scaffolds.

Specimen	AL-Mainframe	AL-Single-Story	AL-Two-Story	AL-Three-Story
Material	Aluminum			
Height	1918 mm	1918 mm	3836 mm	5048 mm
Width	698 mm	1910 mm × 698 mm	1910 mm × 698 mm	1910 mm × 698 mm

**Table 13:** Boundary conditions of structural analysis model.

	x	y	z	xθ	yθ	zθ
Connection boundary conditions	O	O	O	O	O	O
Support boundary conditions	O	O	O	X	X	X

**Table 14:** Material standards for fabricated mobile scaffold.

Members of Scaffold	Component			Material
Mainframe Or Vertical Member	Steel	Column, lateral, and reinforcing member		Equivalent to STK 400 specified in KS D 3566
	Aluminum	Column, lateral, and reinforcing member		Equivalent to A 6063S specified in KS D 6759
Caster Wheel	Spindle and axle			Equivalent to SS400 specified in KS D 3503
	Fork			Equivalent to SPHC specified in KS D 3501
	Tire			Rubber material for products specified in KS B ISO 22877
Guard Rail	Jam band			Equivalent to SS 330 specified in KS D 3503
	Steel	Post, handrail, and brace member		Equivalent to STK 400 specified in KS D 3566
	Aluminum	Post, handrail, and brace member		Equivalent to A 6063S specified in KS D 6759
	Ironware for installation			Equivalent to SPHC specified in KS D 3501 or SS 330 specified in KS D 3503
Outrigger	Steel	Vertical, horizontal, diagonal, reinforcing member, and insertion tube		Equivalent to STK 400 specified in KS D 3566
	Aluminum	Vertical, horizontal, diagonal, reinforcing member, and insertion tube		Equivalent to A 6063S specified in KS D 6759
	Ironware for attachment			Equivalent to SPHC specified in KS D 3501 or Equivalent to SS 330 specified in KS D 3503
Work Plate	Steel	Flooring member		Equivalent to SPHC specified in KS D 3501 or XS 42 specified in KS D 3601
		Horizontal and beam member		Equivalent to SPHC specified in KS D 3501
		Hanging hook	Single plate	Equivalent to SS400 specified in KS D 3503
			Box	Equivalent to SPHC specified in KS D 3501
	Aluminum	Flooring, horizontal, and beam member		Equivalent to A 6063S specified in KS D 6759
		Hanging hook	Single plate	Equivalent to A 5052P specified in KS D 6701 or A 6063S specified in KS D 6759
			Box	Equivalent to A 5052P specified in KS D 6701 or A 6063S specified in KS D 6759

**Table 15:** Example of maximum load according to the work class for vertical load and number of steps of the working platform.

Class	Vertical Load per Unit Area (kN/m^2^)	Performance Certification for Vertical Load (kN)		
		Number of Work Plates		
		1	2	3
1	1.25	8	16	24
2	2.5	15	30	45
3	3.5	21	41	61

**Table 16:** Example of safety certification standards for horizontal loads acting on the fabricated mobile scaffold for limiting horizontal displacement.

Class	Number of Work Plate		
	1	2	3
1	0.4	0.8	1.2
2	0.75	1.5	2.25
3	1.1	2.1	3.1

**Table 17:** Specimens.

Specimen	AL-1	AL-3	ST-1
Shape	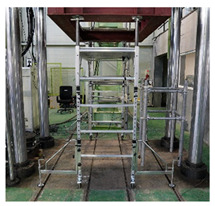	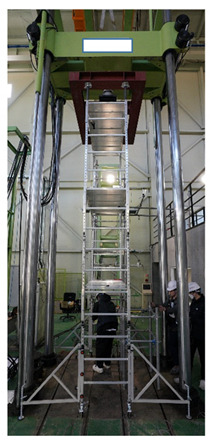	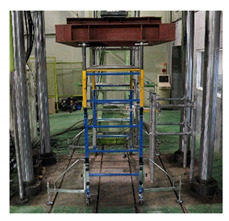
Note	1-story aluminum	3-story aluminum	1-story steel
Height	1918 mm	5048 mm	1918 mm
Width	1910 mm × 698 mm	1910 mm × 698 mm	1910 mm × 698 mm

**Table 18:** Calculation of suggested safety certification standards for fabricated mobile scaffold for vertical loads based on specimens.

Class	Vertical Load per Unit Area (kN/m^2^)	Performance Certification for Vertical Load (kN)		
		Number of Work Plates		
		1	2	3
1	1.25	5.42	10.84	16.26
2	2.5	10.09	20.18	30.27
3	3.5	13.83	27.66	41.48

**Table 19:** Construction of the work class of the specimen based on the experimental results.

Specimen	Materials	Number of Work Plates	Ultimate Load (kN)	Application Class
ST-1	Steel	1	43.8	3
AL-1	Aluminum	1	31.8	2
AL-3		3	43.4	3

**Table 20:** Calculation of suggested safety certification standards for fabricated mobile scaffold for horizontal loads based on specimens.

Class	Vertical Load per Unit Area (kN/m^2^)	Performance Certification for Vertical Load (kN)		
		Number of Work Plates		
		1	2	3
1	1.25	0.27	0.54	0.81
2	2.5	0.50	1.01	1.51
3	3.5	0.69	1.383	2.07

## Data Availability

Data are contained within the article.
